# A combined index to classify prognostic comorbidity in candidates for radical prostatectomy

**DOI:** 10.1186/1471-2490-14-28

**Published:** 2014-03-29

**Authors:** Michael Froehner, Anna-Elisa Kellner, Rainer Koch, Gustavo B Baretton, Oliver W Hakenberg, Manfred P Wirth

**Affiliations:** 1 Departments of Urology, University Hospital “Carl Gustav Carus”, Technische Universität Dresden, Dresden, Fetscherstrasse 74, D-01307 Dresden, Germany; 2 Department of Medical Statistics and Biometry, University Hospital “Carl Gustav Carus”, Technische Universität Dresden, Dresden, Fetscherstrasse 74, D-01307 Dresden, Germany; 3 Department of Pathology, University Hospital “Carl Gustav Carus”, Technische Universität Dresden, Fetscherstrasse 74, D-01307 Dresden, Germany; 4 Department of Urology, University of Rostock, Ernst-Heydemann-Strasse 6, D-18055 Rostock, Germany

**Keywords:** Prostate cancer, Radical prostatectomy, Comorbidity, Overall survival, Competing mortality, ASA classification, Charlson score, Body mass index, Cox proportional hazard models

## Abstract

**Background:**

In patients with early prostate cancer, stratification by comorbidity could be of importance in clinical decision making as well as in characterizing patients enrolled into clinical trials. In this study, we investigated several comorbidity classifications as predictors of overall mortality after radical prostatectomy, searching for measures providing complementary prognostic information which could be combined into a single score.

**Methods:**

The study sample consisted of 2205 consecutive patients selected for radical prostatectomy with a mean age of 64 years and a mean follow-up of 9.2 years (median: 8.6). Seventy-four patients with incomplete tumor-related data were excluded. In addition to age and tumor-related parameters, six comorbidity classifications and the body mass index were assessed as possible predictors of overall mortality. Kaplan-Meier curves and Mantel-Haenszel hazard ratios were used for univariate analysis. The impact of different causes of death was analyzed by competing risk analysis. Cox proportional hazard models were calculated to analyze combined effects of variables.

**Results:**

Age, Gleason score, tumor stage, Charlson score, American Society of Anesthesiologists (ASA) physical status class and body mass index were identified a significant predictors of overall mortality in the multivariate analysis regardless whether two-sided and three-sided stratifications were used. Competing risk analysis revealed that the excess mortality in patients with a body mass index of 30 kg/m^2^ or higher was attributable to competing mortality including second cancers, but not to prostate cancer mortality.

**Conclusion:**

Stratifying patients by a combined consideration of the comorbidity measures Charlson score, ASA classification and body mass index may assist clinical decision making in elderly candidates for radical prostatectomy.

## Background

Because of the usually slow disease progression and the competing curative treatment options with different impacts on quality of life, comorbidity is of particular clinical importance in men with early prostate cancer [1,2]. There is, however, no consensus on the best comorbidity classification to use in this situation [3-5]. The Charlson score [6] has probably been most extensively studied [4,5,7]. In addition, a multitude of other assessment instruments have been evaluated with, however, inconclusive results [3,5]. The complementary prognostic value of different comorbidity classifications has – to our knowledge – not been demonstrated yet in patients with early prostate cancer. Stratifying by comorbidity would be important in clinical decision making as well as in the characterization of patients enrolled into clinical trials. In this study, we investigated several comorbidity classifications as predictors of overall mortality after radical prostatectomy, searching for measures providing complementary prognostic information which could be combined into a single score.

## Methods

### Study sample

The study sample consisted of all 2205 patients who underwent radical prostatectomy between December 1st, 1992 and December 31st, 2005 at our institution (a university hospital). Approval by the institutional review board of the University Hospital Dresden was obtained (approval reference: EK 268092009). Seventy-four patients with missing data on Gleason score, local tumor stage or lymph node status were excluded thus leaving 2131 patients for analysis. Further demographic data is given in Tables 1 and 2.

**Table 1:** The results of the univariate analyses using two-sided stratifications

**Category**	**Events**	**Hazard ratio**	**95% CI**	**p**	**10-year survival**	**95% CI**
PSA < 10 ng/mL	138/1165	1			87.3%	84.7-89.4%
PSA 10+ ng/mL or neoadjuvant therapy	163/966	1.18	0.93-1.48	0.1665	83.5%	80.5-86.0%
Gleason score <8	198/1710	1			88.4%	86.4-90.1%
Gleason score 8-10	103/421	3.44	2.55-4.66	<0.0001	72.5%	66.7-77.4%
organ confined	168/1424	1			88.1%	85.9-90.0%
non confined	133/707	1.64	1.29-2.09	<0.0001	80.1%	76.3-83.3%
pN0	248/938	1			87.2%	85.2-88.9%
pN1	53/193	2.85	1.92-4.23	<0.0001	67.9%	59.2-75.1%
ASA 1-2	218/1774	1			87.9%	85.9-89.6%
ASA 3	83/357	2.88	2.09-3.98	<0.0001	73.3%	67.2-78.4%
Charlson score 0-1	220/1809	1			87.6%	85.7-89.4%
Charlson score 2+	81/322	3.18	2.28-4.43	<0.0001	73.0%	66.7-78.3%
NYHA 0-1	270/2002	1			85.8%	83.8-87.6%
NYHA 2+	31/129	1.91	1.21-3.03	0.0059	79.2%	70.4-85.7%
CCS 0-1	268/2015	1			86.4%	84.5-88.1%
CCS 2+	33/116	2.85	1.74-4.67	<0.0001	71.2%	60.7-79.4%
Disease count 0-1	145/1296	1			89.1%	86.8-91.0%
Disease count 2+	156/835	1.97	1.55-2.49	<0.0001	79.7%	76.2-82.8%
No diabetes with end organ damage	276/2034	1			86.2%	84.3-87.9%
Diabetes with end organ damage	25/97	3.19	1.78-5.71	0.0001	68.3%	55.2-78.3%
Body mass index <30 kg/m^2^	237/1775	1			86.4%	84.4-88.3%
Body mass index 30+ kg/m^2^	64/356	1.69	1.22-2.32	0.0014	80.4%	75.0-84.8%

CI: confidence interval; p values are raw values.

**Table 2:** The results of the univariate analyses using three-sided stratifications

**Category**	**Events**	**Hazard ratio**	**95% CI**	p*	**10-year survival**	**95% CI**
PSA < 10 ng/mL	138/1165	1			87.3%	84.7-89.4%
PSA 10–19.9 ng/mL	58/398	1.03	0.76-1.41	0.8429	86.0%	81.4-89.5%
PSA 20+ ng/mL or neoadjuvant therapy	105/598	1.26	0.97-1.64	0.0852	81.9%	77.9-85.2%
Gleason score <7	117/989	1			88.8%	86.3-90.9%
Gleason score 7	81/721	1.09	0.82-1.45	0.5600	87.7%	84.1-90.5%
Gleason score 8-10	103/421	3.19	2.35-4.34	<0.0001	72.5%	66.7-77.4%
organ confined, pN0	162/1380	1			88.3%	86.1-90.2%
non confined, pN0	86/558	1.30	0.99-1.70	0.0637	84.4%	80.4-87.7%
pN1	53/193	3.14	2.09-4.72	<0.0001	67.9%	59.2-75.1%
ASA 1	17/212	1			92.9%	88.0-95.9%
ASA 2	201/1561	1.83	1.27-2.65	0.0013	87.1%	84.9-89.0%
ASA 3	83/357	3.28	2.18-4.91	<0.0001	73.3%	67.2-78.4%
Charlson score 0	146/1323	1			89.4%	87.3-91.2%
Charlson score 1	74/486	1.53	1.13-2.08	0.0057	82.8%	78.0-86.6%
Charlson score 2+	81/322	3.61	2.56-5.09	<0.0001	73.0%	66.7-78.3%
NYHA 0	245/1877	1			86.4%	84.4-88.2%
NYHA 1	25/125	1.84	1.11-3.06	0.0181	77.0%	67.0-84.4%
NYHA 2+	31/129	2.01	1.26-3.22	0.0034	79.2%	70.4-85.7%
CCS 0	228/1804	1			87.2%	85.2-89.0%
CCS 1	40/211	1.65	1.12-2.45	0.0123	79.5%	71.9-85.2%
CCS 2+	33/116	3.11	1.87-5.16	<0.0001	71.2%	60.7-79.4%
Disease count 0	64/619	1			91.2%	88.2-93.4%
Disease count 1	81/677	1.25	0.90-1.73	0.1822	87.2%	83.6-90.0%
Disease count 2+	156/835	2.00	1.53-2.61	<0.0001	79.7%	76.2-82.8%
No diabetes	249/1877	1			86.7%	84.7-88.4%
Diabetes without end organ damage	27/157	1.47	0.93-2.32	0.0990	80.0%	70.5-86.6%
Diabetes with end organ damage	25/97	3.35	1.86-6.04	0.0001	68.3%	55.2-78.3%
Body mass index <30 kg/m^2^	237/1775	1			86.4%	84.4-88.3%
Body mass index 30–34.9 kg/m^2^	56/327	1.55	1.11-2.16	0.0094	81.4%	75.8-85.8%
Body mass index 35+ kg/m^2^	8/29	6.90	2.10-22.68	0.0015	69.8%	46.1-84.7%

*versus lowest risk category; CI: confidence interval; p values are raw values.

### Investigated variables

Prostate-specific antigen (PSA), Gleason score, tumor stage, Charlson score [6], American Society of Anesthesiologists (ASA) physical status class [8], New York Heart Association (NYHA) class of cardiac insufficiency [9], Canadian Cardiovascular Society (CCS) class of angina pectoris [10], number of concomitant diseases (disease count), diabetes mellitus, and body mass index were investigated as categorical variables. Age was treated as a continuous variable. Patients with neoadjuvant treatment and, therefore, uncertain preoperative PSA values were included in the highest PSA risk groups.

### Data collection

Data was obtained from the patient records. The specimens of patients who underwent surgery prior to 1999 were reclassified in order to ascertain data uniformity. Perioperative cardiopulmonary risk assessment (ASA, NYHA, CCS) classifications were derived from the anesthesiology premedication records. In cases with obviously incorrect classifications these were corrected under the surveillance of a senior anesthesiologist before being entered into a database.

The Charlson score was assigned based on the comorbidity data available in the database supplemented by information derived from the discharge letters largely following the original description of this comorbidity index [6]. The presence of diabetes mellitus with or without end organ damage was recorded separately as another comorbidity classification. Codes for each condition contributing to the Charlson score [6] were included in the database. A disease count was calculated by adding one point for any concomitant disease recorded in our database (angina pectoris, hypertension, history of thrombembolism, body mass index 30 kg/m^2^ or higher, history of myocardial infarction, cardiac insufficiency, peripheral vascular disease, cerebrovascular disease, lung disease, ulcer disease, mild liver disease, diabetes mellitus, connective tissue disease, hemiplegia, moderate or severe renal disease, solid tumors, leukemia, lymphoma, moderate or severe liver disease, dementia, metastatic solid tumors). This was done regardless of the severity of each condition analogous to an approach described by Houterman and co-workers [11]. Follow-up data were collected from urologists and/or general practitioners, the patients, relatives, health insurance companies, local authorities or the local tumor register, whichever was necessary. Thereby, only one patient was lost to follow-up. Causes of death were assigned to the relevant categories by a senior urologist (MF). Prostate cancer was considered the cause of death in cases with uncontrolled disease progression. Second cancers were considered the cause of death when an uncontrolled second malignancy was present at the time of death. Deaths in the absence of uncontrolled prostate or second cancer were considered from deaths from non-cancer competing (“comorbid”) causes. Deaths from accidents or suicide were considered a separate category. The cause of death was identified reliably in all deceased patients.

### Variables and stratifications

Comorbidity variables were included into the analysis when they were available in our database and allowed for a stratification by the degree of severity into 3 categories (none, mild or severe). Two- and three-sided stratifications were investigated for each variable. The stratifications used are shown in Tables 1 and 2. Commonly used stratifications promising a maximal contrast for each parameter were chosen in order not to miss potentially relevant prognostic information.

### Statistical analysis

Overall mortality was the primary study endpoint. Kaplan-Meier curves and Mantel-Haenszel hazard ratios were calculated. Univariate comparisons were made with the log rank test. Only parameters significantly associated with mortality in univariate analysis were used for multivariate analysis. Cox proportional hazard models were calculated to analyze combined effects of variables. Only parameters that remained significantly associated with mortality upon multivariable analysis were retained in the final models. After stratification by body mass index (<30 kg/m^2^ versus higher), the contribution of different causes of death (prostate cancer: uncontrolled recurrent disease, competing causes: all causes of death other than uncontrolled recurrent prostate cancer, non-cancer competing causes: all non-cancer causes other than accidents or suicide, second cancers: all cancer deaths other than from uncontrolled recurrent prostate cancer) was determined by competing risk analysis [12]. The statistical analyses were performed with the Statistical Analysis Systems (SAS Institute, Cary, NC) statistical package.

## Results

**Figure 1:**
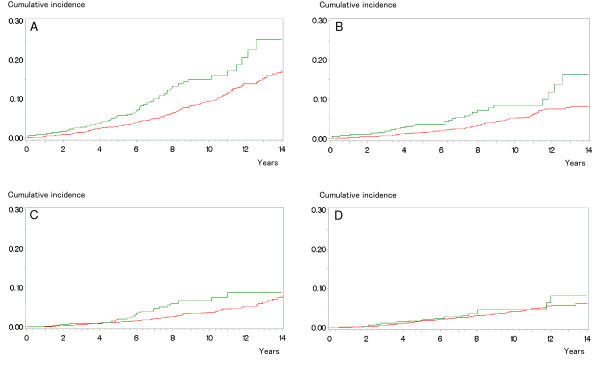
**Competing risk analysis of different causes of death stratified by the body mass index (green: body mass index lower than 30 kg/m**^**2**^**, red: body mass index 30 kg/m**^**2 **^**or higher), A: competing causes altogether (all causes other than prostate cancer), Pepe-Mori test: p = 0.0196, B: non-cancer competing causes (all causes other than prostate or second cancers), Pepe-Mori test: p = 0.0411, C: second cancers, Pepe-Mori test: p = 0.11, D: prostate cancer, Pepe-Mori test: p = 0.69.**

**Figure 2:**
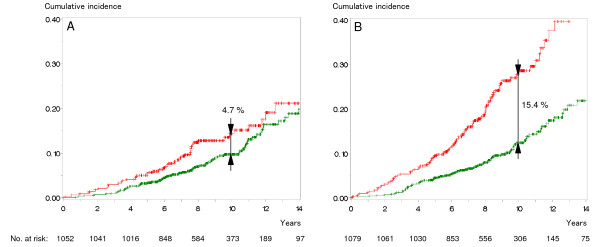
**Cumulative incidence of overall mortality in patients without one of the risk factors ASA 3, Charlson score 2 or higher or body mass index 30 kg/m**^**2 **^**or higher (green) versus those with one of these risk factors (red). A**: patients younger than 65 years (n = 1052), hazard ratio 3.66 (95% confidence interval 1.80-7.43, log rank test: p = 0.0003), **B**: patients aged 65 years or older (n = 1079): hazard ratio 4.27 (95% confidence interval 2.65-6.86, log rank test: p < 0.0001).

The mean age was 64.2 years. The mean follow-up in the surviving patients was 9.2 years (median: 8.6 years; interquartile range 4.3 years). Of the 2131 patients included in the analysis, 84 patients had died of prostate cancer and 227 of competing causes up to now (maximum follow-up: 19.4 years). The results of univariate analyses are shown in Table 1 (two-sided stratifications) and Table 2 (three-sided stratifications) and the optimal Cox proportional hazard models for two-sided and three-sided stratifications are shown in Table 3. In addition to patient age, tumor stage and Gleason score, the Charlson score, the ASA classification and the body mass index provided complementary information on overall survival probability in two-sided as well as in three-sided stratifications (Table 3). Competing risk analysis showed that the excess mortality in obese patients (body mass index of 30 mg/m^2^ or higher) was attributable to competing mortality. This competing mortality was attributable to non-cancer causes as well as second cancers (Figure 1). There was no detectable association between obesity and prostate cancer mortality (Figure 1). Considering patients with and without one of the risk factors Charlson score 2 or higher, the ASA class 3 or body mass index 30 mg/m^2^ or higher, the observed survival difference was somewhat higher in patients aged 65 years or older (Figure 2). Stratifying patients by combining the three comorbidity classifications with complementary prognostic information (weighing was done by adding one for the risk classes ASA 2, Charlson score 1, body mass index 30 kg/m^2^ or higher and two points for the risk classes ASA 3 or Charlson score 3 each patient) resulted in a wide separation of the survival curves with a relatively balanced distribution of the patients over the risk groups particularly when patients aged 65 years or older were considered (Table 4).

**Table 3:** Multivariate analysis: optimal Cox proportional hazard models for two-sided and three-sided stratifications

**Optimal model, 2-sided stratifications**			
**Category**	**Hazard ratio**	**95% confidence interval**	**p**
Age (per year)	1.06	1.04-1.08	<0.0001
Gleason score 8+	2.25	1.74-2.90	<0.0001
pN1	1.70	1.24-2.34	0.0010
ASA 3	1.58	1.17-2.12	0.0030
Charlson score 2+	1.71	1.27-2.30	0.0004
Body mass index 30+ kg/m^2^	1.47	1.10-1.95	0.0080
**Optimal model, 3-sided stratifications**			
**Category**	**Hazard ratio**	**95% confidence interval**	**p**
Age (per year)	1.06	1.03-1.08	<0.0001
Gleason score 7	1.07	0.80-1.43	0.6641
Gleason score 8+	2.21	1.63-3.00	<0.0001
ASA 2	1.84	1.10-3.07	0.0202
ASA 3	2.69	1.50-4.83	0.0009
Charlson score 1	1.24	0.93-1.66	0.1412
Charlson score 2+	1.79	1.29-2.47	0.0004
pT3-4 and pN0	1.07	0.81-1.42	0.6420
pN1	1.85	1.30-2.62	0.0006
Body mass index 30–34.9 kg/m^2^	1.37	1.02-1.84	0.0385
Body mass index 35+ kg/m^2^	1.95	0.94-4.02	0.0718

Reference categories were other patients in the case of two-sided comparisons and the lowest risk category when two-sided comparisons were used.

**Table 4:** Proportion of events, Mantel-Haenszel hazard ratios, 10-year overall survival rates, confidence intervals and p values stratified by the score based on the three comorbidity classifications with complementary information content ASA class, Charlson score and body mass index in the whole sample and subdivided into age groups

**Age <65 years (n = 1052)**						
**Points**	**Events**	**Hazard ratio**	**95% CI**	p*	**10-year survival**	**95% CI**
0	12/137	1			91.5%	84.7-95.4%
1	47/450	1.44	0.81-2.56	0.2133	90.5%	86.7-93.3%
2	23/253	1.30	0.65-2.57	0.4582	90.9%	85.4-94.4%
3	22/125	2.70	1.35-5.39	0.0049	80.3%	69.6-87.6%
4	11/61	2.68	1.07-6.70	0.0346	82.5%	69.8-90.2%
5	6/26	7.11	1.73-29.24	0.0066	74.2%	47.2-88.8%
**Age 65+ years (n = 1079)**						
**Points**	**Events**	**Hazard ratio**	**95% CI**	p*	**10-year survival**	**95% CI**
0	3/53	1			98.1%	87.4-99.7%
1	56/469	2.24	1.08-4.64	0.0300	88.0%	83.6-91.2%
2	46/259	2.60	1.32-5.13	0.0058	80.5%	73.5-85.8%
3	30/157	3.14	1.48-6.69	0.0030	78.2%	69.0-84.9%
4	29/106	4.07	1.98-8.37	0.0001	67.0%	54.7-76.6%
5	16/35	21.12	7.45-59.90	<0.0001	48.9%	26.2-68.3%
**All patients (n = 2131)**						
**Points**	**Events**	**Hazard ratio**	**95% CI**	p*	**10-year survival**	**95% CI**
0	15/190	1			93.4%	88.3-96.3%
1	103/919	1.73	1.11-2.69	0.0149	89.3%	86.5-91.5%
2	69/512	2.00	1.26-3.18	0.0032	85.7%	81.4-89.0%
3	52/282	2.89	1.77-4.71	<0.0001	78.9%	72.2-84.2%
4	40/167	3.88	2.25-6.70	<0.0001	73.0%	64.2-80.0%
5	22/61	21.30	8.95-50.72	<0.0001	60.3%	43.2-73.7%

*versus lowest risk category; CI: confidence interval; p values are raw values.

## Discussion

Overdiagnosis and overtreatment are crucial issues in the management of early prostate cancer. Classifying comorbidity could be a strategy to tackle this problem [13]. However, so far, no consensus on the best way to measure comorbidity in men with early prostate cancer has been reached. Although the guideline of the European Association of Urology mentions the ASA classification beside the Charlson score is as a decision tool [14], to our knowledge, a complementary information content of these two classifications has not yet been demonstrated. Our analysis showed that in men who are candidates for radical prostatectomy the Charlson score, the ASA classification and the body mass index measured different aspects of the health status. Whereas the Charlson score is calculated as a sum of several prognostically relevant diseases with different weights, the ASA classification evaluates the general health status focused on the perioperative risk. Therefore, the observed complementary information content of both classifications (Table 3) was a plausible result. With the exception of the body mass index, all other comorbidity classifications used in our study were more or less related either to the ASA classification or to the Charlson score or both and for that reason did not provide significant complementary information despite a partially strong association with survival in the univariate analysis (Tables 1, 2 and 3).

The ability of a combination of prognostic tools to meaningfully stratify the relatively healthy candidates for radical prostatectomy was clearly superior to that of all investigated comorbidity assessment tools on their own. The combination of the tools resulted in higher survival differences, more balanced distribution of the patients over the risk groups and the consideration of different aspects of the health status. There is currently no other comorbidity classification that reaches overall survival differences between the best and the worst risk group up to roughly 50% (Table 4) in this long-living population. Even in the presence of serious comorbidity, the majority of men selected for radical prostatectomy survive more than 10 years after surgery [15]. With the six risk groups, a relatively balanced distribution of patients, a dose–response relationship, considerable survival differences (Table 4) and easy applicability, this combined comorbidity assessment tool could be useful both for risk stratification in clinical trials and decision making. The combined comorbidity index may be used to counsel candidates for radical prostatectomy with low risk tumors who consider deferred curative treatment or to stratify patients enrolled into clinical trials comparing different treatment options for localized prostate cancer.

Obesity is associated with excess cardiovascular and cancer mortality [16-18]. In men with early prostate cancer, however, conflicting data have been reported about the prognostic significance of the body mass index [19,20]. In our study, we found a significant association of obesity (defined as a body mass index of 30 kg/m^2^ or higher) with competing mortality but not with prostate cancer mortality after radical prostatectomy (Figure 1). This observation is supported by the relatively small contribution of prostate cancer to excess cancer mortality in obese men compared with that of several other neoplasms [17]. Thus, due to this association of obesity and excess competing (but not prostate cancer) mortality, the body mass index appears to be a suitable predictor of competing mortality complementary to the other comorbidity measures in candidates for radical prostatectomy.

The vast majority of patients selected for radical prostatectomy belong to the low risk group in the Charlson score (Charlson score 0: in our study 62%; elsewhere: 73% [4]) and to the intermediate risk group in the ASA classification (ASA 2: in our study 73%; elsewhere: 65% [21]). These imbalances limit the applicability of both classifications on their own in clinical decision making. In contrast, the combination of the Charlson score, the ASA classification and the body mass index distributed the patients in fairly balanced way over even more risk groups (Table 4). Furthermore, the combination of a fairly subjective assessment as the ASA classification with more objective instruments as the Charlson score and the body mass index compensates for the intrinsic weaknesses of each classification, i. e. the lack of recording specific comorbidities in the ASA classification and the disregard of the personal impression of the patient’s health status with the Charlson score and the body mass index. The simultaneous recording of all three indicators may also allow for a solution of the problem of retrospectively determining the severity of health problems with chart review and may compensate for errors in classification. All three comorbidity measures are widely used and easily applicable, even though the assignment of the ASA class required a visit by a sufficiently experienced anesthesiologist.

This study has several limitations. Although the mean follow-up was relatively long, only a small number of patients died during follow-up. It is not entirely sure that these patients are representative for the whole sample. Furthermore, patient recruitment in our study started about 20 years ago. Over that period of time, diagnostic methods, classifications and the treatment of concomitant diseases have changed and the life expectancy might have increased. A complementary prognostic impact of the ASA classification and the Charlson score has already been demonstrated for patients with bladder cancer [22,23], but not yet for patients with prostate cancer. Therefore, a confirmation and validation of our findings in different samples would be desirable. Given a sufficient sample size and follow-up, such validation would require limited effort because of the rapid accessibility of the ASA classification and the Charlson score (as well as the body mass index) during retrospective chart review. The survival rates observed in this study are only valid for the population investigated (candidates for radical prostatectomy) but not for different populations (unselected patients or patients selected for other treatment options for localized prostate cancer.

## Conclusion

The three easily applicable comorbidity classifications Charlson score, ASA classification and body mass index measure different prognostic aspects of the health status in candidates for radical prostatectomy. Compared with the use of one of these three classifications on its own, the combination of the three instruments has several advantages. The combined assessment provides a more balanced distribution of patients over the risk groups, stratification into more risk groups, a greater survival difference between the best and worst risk groups and a higher resistance to classification errors by employing different and relatively independent aspects of the health status.

## Abbreviations

PSA: Prostate-specific antigen; ASA: American Society of Anesthesiologists; NYHA: New York Heart Association; CCS: Canadian Cardiovascular Society; SAS: Statistical Analysis Systems; BMI: Body mass index.

## Competing interests

The authors declare no conflicts of interest related to the matter discussed in this manuscript.

## Authors’ contributions

MF designed the study, collected and analysed data, wrote and drafted the manuscript, AEK collected follow-up data, RK performed the statistical analysis and drafted the manuscript, GBB contributed histopathological data, OWH drafted the manuscript, MPW provided administrative support, interpreted the data and drafted the manuscript. All authors read and approved the final manuscript.

## Authors’ information

MF is associate professor at the Department of Urology, AEK is medical student, RK is retired professor at the Department of Medical Informatics and Biometry, GBB is professor and chairman at the Department of Pathology of the Dresden University of Technology, OWH is professor and chairman at the Department of Urology of the University of Rostock, MPW is professor and chairman at the Department of Urology of the Dresden University of Technology.

## Pre-publication history

The pre-publication history for this paper can be accessed here:

http://www.biomedcentral.com/1471-2490/14/28/prepub
